# Wait Times and Survival in Lung Cancer Patients across the Province of Quebec, Canada

**DOI:** 10.3390/curroncol29050259

**Published:** 2022-04-29

**Authors:** Marie-Hélène Denault, Catherine Labbé, Carolle St-Pierre, Brigitte Fournier, Andréanne Gagné, Claudia Morillon, Philippe Joubert, Serge Simard, Simon Martel

**Affiliations:** 1 Centre de Recherche de l’Institut Universitaire de Cardiologie et de Pneumologie de Québec, 2725 Ch. Ste-Foy, Québec, QC G1V 4G5, Canada; catherine.labbe@criucpq.ulaval.ca (C.L.); carollest-pierre@videotron.ca (C.S.-P.); brigitte.fournier@criucpq.ulaval.ca (B.F.); andreanne.gagne.4@ulaval.ca (A.G.); claudia.morillon@usherbrooke.ca (C.M.); philippe.joubert@criucpq.ulaval.ca (P.J.); serge.simard@criucpq.ulaval.ca (S.S.); simon.martel@fmed.ulaval.ca (S.M.); 2 BC Cancer Agency-Vancouver Center, 600 W 10th Avenue, Vancouver, BC V5Z 4E6, Canada

**Keywords:** non-small cell lung cancer, small cell lung cancer, wait times, delays, timeliness, overall survival

## Abstract

Lung cancer is the leading cause of cancer death worldwide, with a five-year survival of 22% in Canada. Guidelines recommend rapid evaluation of patients with suspected lung cancer, but the impact on survival remains unclear. We reviewed medical records of all patients with newly diagnosed lung cancer in four hospital networks across the province of Quebec, Canada, between 1 February and 30 April 2017. Patients were followed for 3 years. Wait times for diagnosis and treatment were collected, and survival analysis using a Cox regression model was conducted. We included 1309 patients, of whom 39% had stage IV non-small cell lung cancer (NSCLC). Median wait times were, in general, significantly shorter in patients with stage III–IV NSCLC or SCLC. Surgery was associated with delays compared to other types of treatments. Median survival was 12.9 (11.1–15.7) months. The multivariate survival model included age, female sex, performance status, histology and stage, treatment, and the time interval between diagnosis and treatment. Longer wait times had a slightly protective to neutral effect on survival, but this was not significant in the stage I–II NSCLC subgroup. Wait times for the diagnosis and treatment of lung cancer were generally within targets. The shorter wait times observed for advanced NSCLC and SCLC might indicate a tendency for clinicians to act quicker on sicker patients. This study did not demonstrate the detrimental effect of longer wait times on survival.

## 1. Introduction

Lung cancer is one of the most diagnosed cancers in both men and women. Found at an advanced stage in 69% of cases, it is the leading cause of cancer-related death worldwide, with a five-year survival of 22% in Canada [1,2,3]. Age, sex, smoking, overall health, performance status, weight loss, histology, presence of driver mutations (for non-small cell lung cancer), and treatment modality have all been shown to influence outcomes [3,4,5,6,7,8,9]. However, the stage at diagnosis remains the most important prognostic factor [6,8], as patients with early-stage disease might be eligible for curative-intent treatment. Investigation and management wait times could indirectly impact outcomes, delays putting the patient at risk of disease upstaging [10]. Furthermore, patient satisfaction with wait times is a central domain of quality improvement, influenced by care coordination, provider interpersonal skills, and timeliness [11]. The latter is defined as “the speed and efficiency with which care is provided, with attempts to avoid or mitigate anticipated delays” and results from actions by the responsible physicians, the system, and the patients [11]. A few societies over the world have published targets for wait times in lung cancer, the most detailed coming from the British Thoracic Society (BTS) [12]. Key recommendations include a maximum delay of 7 days from referral to consultation with a lung cancer specialist, 56 days from this consultation to surgery, and 28 days from surgical listing to surgery [12]. Guidelines from Quebec’s Ministère de la Santé et des Services sociaux (MSSS) recommend that surgery be performed within 28 days of the signature of the operating room requisition by the surgeon in 90% of patients and within 56 days in 100% [13]. For non-surgical treatments, the BTS recommends an interval of 7–28 days between the decision to treat and treatment start (7 days for chemotherapy, 14 days for palliative radiation, and 28 days for radical radiation), while the MSSS states that radiation should start within 56 days of the moment the patient is deemed “ready-to-treat” [14]. Finally, the Research ANd Development (RAND) corporation mentions an interval ≤42 days between diagnosis and first treatment and a diagnosis within 2 months of the first abnormal imaging [15]. Those recommendations are based on expert opinion and consensus since the relationship between wait times and outcomes, although plausible, remains unclear [10,16,17,18]. We conducted a retrospective cohort study to determine the wait times for lung cancer diagnosis and treatment in the province of Quebec, Canada, and to investigate their relationship with survival.

## 2. Materials and Methods

Patients were identified retrospectively through the Quebec Hospitals Cancer Registries. All consecutive patients covered by provincial health insurance with a new diagnosis of lung cancer made between 1 February and 30 April 2017 in the province of Quebec, Canada, were eligible. Patients with lesions that required previous imaging surveillance and patients who did not complete their healthcare trajectory within 4 months of pathological or cytological diagnosis because of comorbidities or noncompliance were excluded. The Institut universitaire de cardiologie et de pneumologie de Québec (IUCPQ), a specialized heart and lung institute in Quebec City, was the coordinating center. Central Research Ethics Board (REB) approval was obtained before the beginning of screening.

**Figure 1:**
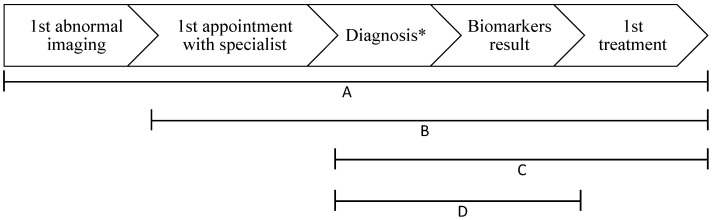
Diagnosis and treatment time intervals. * Defined as the date of the pathology result. A = 1st abnormal imaging to 1st treatment; B = 1st appointment with specialist to 1st treatment. C = diagnosis to 1st treatment (including surgery, definitive radiation, definitive chemoradiation, and systemic treatment); D = pathology result to biomarkers result. A fifth interval, radiation referral to radiation, is not displayed on this figure.

Data collection began on 1 September 2017, 4 months after the end of the inclusion period, to ensure that patients had completed their diagnostic trajectory up to the first treatment, as mentioned above. Two clinical trials nurses and a medical student performed data collection on a standardized case report form. Data were abstracted from patients’ charts at each individual institution and from the provincial deaths registry for survival. Hospitals were aggregated into four networks according to their catchment area: IUCPQ, Alliance Estrie-Montérégie, Centre hospitalier de l’Université de Montréal (CHUM) and Centre universitaire de santé McGill (CUSM). Demographics, histology (grouped as non-small-cell lung cancer (NSCLC) and small cell lung cancer (SCLC)), stage at diagnosis (according to the 8th Edition of the TNM Classification for Lung Cancer [19]), biomarker status, procedures leading to diagnosis and treatments received were collected, as well as dates of abnormal imaging, pathological diagnosis, appointment with a lung cancer specialist, and treatment start. Supportive treatment, palliative radiation, and palliative care consultation were all categorized as “symptom-directed treatment”. The following time intervals were selected and calculated based on relevance to the patient and to evaluate specialist-dependent timeliness (Figure 1):

- First abnormal imaging to first treatment;
- First appointment with a specialist to first treatment;
- First appointment with a specialist to surgery;
- Diagnosis to first treatment;
  ○ Diagnosis to surgery;
  ○ Diagnosis to definitive radiation;
  ○ Diagnosis to definitive chemoradiation;
  ○ Diagnosis to systemic treatment.
- Radiation referral to radiation;
- Diagnosis to biomarker result.

The main outcome was overall survival, defined as the time between diagnosis (pathology report date) and death from all causes. The data cutoff date was 13 May 2020, ensuring a minimum follow-up of 3 years for all included patients.

Descriptive statistics were used for demographic characteristics and wait times. Proportions and either means with standard deviation or medians with range were calculated, respectively, depending on the distribution. Negative wait times, for example, reflecting a pathological diagnosis obtained after treatment start, were excluded as they do not capture the timeliness of care. Patients in the “symptom-directed treatment” category were also excluded from wait times calculations. Comparisons between networks and subgroups were made using chi-square tests for categorical variables and t-tests for continuous variables. A Kaplan–Meier curve was used for the survival analysis. Cox regression was used to test the relationship between wait times and survival, with the exclusion of negative wait times. Since the risk was not proportional over time, and this threshold was around 60 days, separate hazard ratios ≤ 60 days and >60 days were calculated. Subgroup analyses according to the stage (NSCLC I–II versus III–IV) and histology (NSCLC versus SCLC) were performed. Cox regression was again used to test not only wait times but also age, sex, smoking history, performance status, hospital network, histology, stage, and treatment, first in univariate analyses. Based on the variables that had a statistically significant association with survival in univariate analyses, a multivariate model was built. Participants with missing data for certain wait times or survival were excluded from those particular analyses. The statistical significance threshold was *p* < 0.05 for all the analyses.

## 3. Results

**Figure 2:**
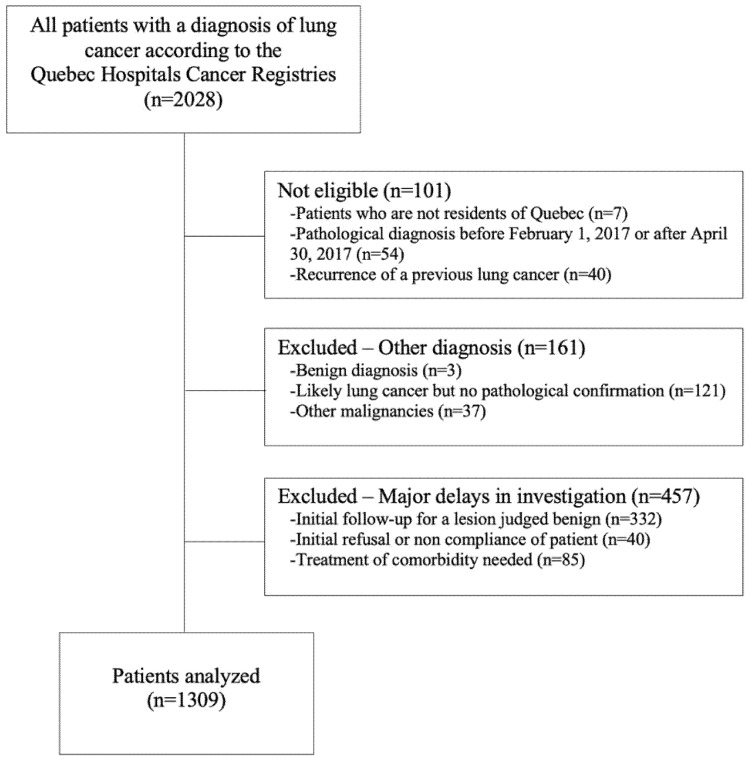
Flow chart of patient selection.

Between 1 February and 31 April 2017, according to the Quebec Hospitals Cancer Registries, there were 2208 patients with a new diagnosis of lung cancer. Of those, 101 were deemed ineligible based on their date of diagnosis or province of residence or because they were presenting with cancer recurrence rather than a new diagnosis. Another 618 were excluded, either because of misdiagnosis, absence of pathological confirmation, or major delays in investigations, leaving a study population of 1309 patients (Figure 2). Patient characteristics are presented by network in Table 1. Most patients belonged to IUCPQ (*n* = 454, 35%) or CHUM (*n* = 441, 34%). Subpopulations by network were comparable for almost every parameter, with a mean age of 68.7 ± 9.2 years, slightly more females (52%) than males (48%), and a vast majority of smokers (91%). Stage IV NSCLC (39%) and adenocarcinoma (52%) were the most represented stage and histology, respectively, and SCLC accounted for 14% of cases. Accordingly, treatment was most frequently systemic (28%) or symptom-directed (26%).

There were significant differences between networks for treatment type, with more patients getting surgery at CHUM versus more getting symptom-directed treatment at IUCPQ, for example. Biomarker status was unknown for most patients. However, among those who were tested, EGFR mutation was found in 7% (30/423), ALK translocation in 1% (6/411), and PD-L1 tumor proportion score ≥ 50% in 39% (156/399). Patients had, on average, seven procedures to obtain a diagnosis, counting imaging and sampling procedures. Flexible bronchoscopy or transthoracic needle biopsy led to diagnosis in most cases (51%). A tumor board review was held for 15% of patients.

Wait times for investigation and treatment are presented in Table 2, Table A1 and Table A2. The median time for the whole diagnostic pathway, from first abnormal imaging to first treatment, was 80 days. Two hundred and ten patients had a negative diagnosis-to-treatment interval. A majority of those had early-stage NSCLC treated with surgery, meaning the pathological diagnosis was made postoperatively with the resected specimen. As specified in the methods, those patients were excluded from wait times calculations.

Surgery seemed to generate delays compared to other treatments, as the median time from the first appointment with a specialist to surgery was 76 days, compared to 57 days for all types of treatments together. Furthermore, a median of 31 days elapsed between diagnosis and treatment, and again, this was longer for surgery (45 days) compared to definitive radiation (40 days), definitive chemoradiation (30 days), and systemic treatment (21 days). Interestingly, wait times were significantly shorter for advanced NSCLC compared to early-stage NSCLC and for SCLC compared to NSCLC (Table A1 and Table A2). Indeed, the median time from diagnosis to treatment was 29 versus 48 days for advanced and early-stage NSCLC, respectively (*p* < 0.0001), and it was 13 days in SCLC compared to 35 days in NSCLC (*p* < 0.0001).

**Figure 3:**
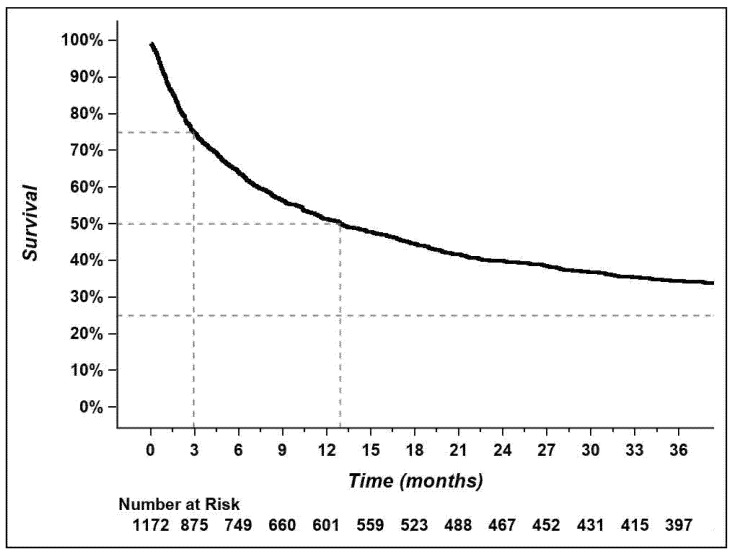
Overall survival for the whole cohort (*n* = 1172). Data cutoff date was 13 May 2020. Overall survival was defined as the time from diagnosis to death.

Overall survival was calculable for 1172 of 1309 patients (90%). Median overall survival was 12.9 (11.1–15.7) months, with a 3-year survival rate of 34.4% (Figure 3). Overall, longer wait times had a slightly protective to neutral effect on survival (Table 3). This relationship remained significant for most analyses after adjustment for possible confounding variables (performance status, histology, stage, and primary treatment).

Subgroup analyses according to stage and histology showed that this proportional relationship between wait times and survival was not significant in early-stage NSCLC and in SCLC (Table A3 and Table A4).

Cox regression identified age, female sex, performance status, histology, stage, primary treatment, and the diagnosis-to-first-treatment interval as significant prognostic factors in the multivariate model (Table 4). The hospital network was not significant. An incremental effect was observed for performance status, histology, and stage, as well as treatment, meaning that worse performance status, small cell histology, higher stage, and less aggressive treatment were more detrimental to survival. Extensive-stage small cell lung cancer (ES-SCLC) had the worse prognosis, with an HR for death of 4.46 compared to stage I NSCLC. Patients on systemic treatment also fared worse than those on definitive chemoradiation, definitive radiation, and surgery (HR for death 2.92 compared to surgery).

## 4. Discussion

This retrospective, multicentric cohort study looked at wait times for the diagnosis and treatment of lung cancer in the province of Quebec, Canada, and investigated their impact on survival. The median wait time from first abnormal imaging to treatment was 80 days. While society guidelines do not have recommendations for this particular interval, the RAND corporation states that the interval between the first abnormal imaging and diagnosis should be less than 2 months [15]. In our study, knowing the first-abnormal-imaging-to-treatment interval (80 days) and the diagnosis-to-first-treatment interval (31 days), the median time from first abnormal imaging to diagnosis was 49 days, well within the recommended delay for at least half of the patients as this is a median. The RAND corporation also recommends a delay of ≤ 42 days between diagnosis and treatment, which was 31 days in the present study. Our median intervals between diagnosis and definitive radiation, definitive chemoradiation, and systemic treatment were 40, 30, and 21 days, respectively, all within the recommended treatment-specific wait times of 42 days from the same society. Surgery, on the other hand, was associated with the longest interval between diagnosis and treatment (45 days), probably reflecting the additional investigations required for preoperative evaluation [18,20,21]. The median wait time between consultation with a lung specialist and surgery was 76 days, failing to meet the BTS target of 56 days [12]. This excessive delay is cause for concern considering the risk of disease upstaging, which could jeopardize eligibility for potentially curative treatment.

Subgroup analyses demonstrated a general tendency toward shorter wait times in SCLC and advanced (stage III–IV) NSCLC compared to early-stage NSCLC. This finding could illustrate the clinical reflex to act quicker on sicker patients [18,20], as well as the higher likelihood of getting systemic treatment, which does not require as much planning.

The median overall survival was 12.9 months, and the 3-year survival rate was 34.4%. Those numbers align with the most recent data reported by the Canadian Cancer Society, based on cases diagnosed between 2015 and 2017 and excluding the province of Quebec, however [3].

Longer wait times had a slightly protective to neutral effect on survival, which is not surprising considering the many other factors at play. It is logical to think that timely investigation and treatment would have a positive impact on outcomes, but we were not able to capture this in the present study. Nonetheless, a reassuring finding is that this slightly protective association between longer wait times and survival became non-significant in the stage I–II NSCLC subgroup, perhaps suggesting that timeliness does matter in curable disease.

The most significant variables in our multivariate analysis (age, female sex, performance status, stage, histology) were already well-known prognostic factors in the literature [3,4,5,6,7,8,9]. The incremental trend in the relationship with survival was imperfect for histology and stage as well as treatment. Indeed, the HR for death was higher for NSCLC stage III (HR 3.92) compared to stage IV (HR 3.61) and for definitive radiation (HR 1.67) compared to definitive chemoradiation (HR 1.61). However, the confidence intervals around the hazard ratios were overlapping. The incremental trend was not as pronounced in multivariate compared to univariate analyses, perhaps because of potential collinearity between ECOG, stage, and treatment, although this was not formally tested.

The results of this study are similar to those observed in previous retrospective cohort studies looking at wait times and survival in lung cancer [20,21,22]. Largey and al. performed a monocentric audit of 126 patients newly diagnosed with lung cancer; the diagnosis-to-treatment interval was 30.4 days, very close to the 31 days we observed [21]. Looking at wait times but also survival, Kasymjanova et al. included 751 patients from one center diagnosed with lung cancer between 2010 and 2014. The authors found wait times similar to ours for the specialist-to-treatment (54 days) and diagnosis-to-treatment (27 days) intervals [20]. The 12-month survival rate was better in that study (56%) compared to ours (38%), but patients with SCLC and supportive treatment were excluded from the survival analysis in the former. A diagnosis-to-treatment interval ≤ 30 days was associated with better survival, but this relationship was reversed for advanced stages, similar to the slightly protective effect of longer wait times we found in our study. The two aforementioned studies, as well as others, have identified SCLC, advanced stage, and non-surgical treatment as factors associated with shorter diagnosis and management wait times [20,21,22,23], similar to what we observed. Possible underlying reasons might be that patients with SCLC or advanced disease are more likely to be symptomatic and, thus, prompt quicker management. Moreover, as previously mentioned, non-surgical treatment requires less investigation and planning, which could explain a shorter interval of treatment.

The meticulous screening and inclusion of all eligible patients is a strength of this study. We also selected the intervals that seemed the most relevant to both patients and clinicians. The survival model tested not only those wait times but also known prognostic factors for which data had been collected, making the final model as thorough as possible. This study presents real-world data from both academic and community hospitals, making the results potentially generalizable to similar healthcare systems.

The main limitation comes from losses to follow-up and missing data. The overall survival estimate is still based on 90% of our sample and is thus representative of the survival of the whole cohort. The remaining 10% could not be censored as the only possible time point after diagnosis would have been the date of the first treatment, and this short interval would have led to an underestimation of survival. A high number of patients with negative diagnosis-to-treatment intervals and/or supportive treatment were excluded as well, resulting in a multivariate survival model based on 761/1309 patients. This model succeeded in identifying prognostic factors that are well-established in the literature, which is a reassuring finding. As for the relationship between wait times and survival, we aimed to mitigate potential dilution of the association by excluding negative wait times and patients who received supportive care, but a type II error remains possible.

## 5. Conclusions

In conclusion, the lung cancer investigation and management wait times in the province of Quebec, Canada, generally meet the targets established by international guidelines. Surgery generates delays compared to other treatment modalities, and the interval between consultation with the lung cancer specialist and resection is still too long. While part of this is certainly attributable to preoperative evaluation, efforts should be made to better understand and modify the factors involved in those delays in order to offer potentially curative treatment to the greatest number of patients. This study did not demonstrate a detrimental effect of diagnosis and treatment delays on survival, but this should be interpreted with caution considering the missing data. Regardless of our findings and their limitations, timeliness of care remains important as it carries the potential advantages of sparing healthcare resources and contributing to the satisfaction of both the patient and the cancer care team.

## Figures and Tables

**Table 1:** Patient characteristics by hospital network (*n* = 1309).

	Alliance Estrie-Montérégie (*n* = 214, 16%)	CHUM (*n* = 441, 34%)	CUSM (*n* = 200, 15%)	IUCPQ (*n* = 454, 35%)	*p*-Value
Age, y	68.8 ± 8.4	68.0 ± 9.2	69.1 ± 9.4	68.1 ± 9.7	0.5
Sex					0.3
Male	114 (53%)	209 (47%)	90 (45%)	213 (47%)	
Female	100 (47%)	232 (53%)	110 (55%)	241 (53%)	
Smoking status					0.04
Ever-smoker	200 (94%)	409 (93%)	172 (86%)	416 (92%)	
Never-smoker	5 (2%)	21 (5%)	18 (9%)	22 (5%)	
Unknown	9 (4%)	11 (3%)	10 (5%)	16 (4%)	
ECOG performance status					<0.0001
0	56 (26%)	173 (39%)	53 (27%)	160 (35%)	
1	57 (27%)	117 (27%)	53 (27%)	159 (35%)	
2	54 (25%)	74 (17%)	49 (25%)	82 (18%)	
3	34 (16%)	61 (14%)	26 (13%)	35 (8%)	
4	1 (1%)	2 (1%)	2 (1%)	0	
Unknown	12 (6%)	14 (3%)	17 (9%)	18 (4%)	
Histopathology					0.1
NSCLC					
Adenocarcinoma	120 (56%)	240 (54%)	105 (53%)	222 (49%)	
Squamous cell	42 (20%)	96 (22%)	46 (23%)	112 (25%)	
Other ^§^	12 (6%)	51 (12%)	20 (10%)	55 (12%)	
SCLC	40 (19%)	54 (12%)	29 (15%)	65 (14%)	
Stage					0.7
NSCLC					
I	44 (21%)	99 (23%)	42 (21%)	92 (20%)	
II	15 (7%)	32 (7%)	13 (7%)	40 (9%)	
III	32 (15%)	89 (20%)	40 (20%)	77 (17%)	
IV	83 (39%)	167 (38%)	76 (38%)	180 (40%)	
SCLC					
Limited	10 (5%)	15 (3%)	4 (2%)	16 (4%)	
Extensive	30 (14%)	39 (9%)	25 (13%)	49 (11%)	
Biomarkers					
EGFR mutation					
Positive	8 (4%)	13 (3%)	4 (2%)	5 (1%)	0.02
Negative	66 (31%)	150 (34%)	62 (31%)	115 (25%)	
Unknown	140 (65%)	278 (63%)	134 (67%)	334 (74%)	
ALK translocation					
Positive	1 (1%)	3 (1%)	1 (1%)	1 (0%)	0.02
Negative	71 (33%)	165 (37%)	54 (27%)	121 (27%)	
Unknown	142 (66%)	273 (62%)	145 (73%)	332 (73%)	
PD-L1 expression					
<1%	10 (5%)	32 (7%)	25 (13%)	18 (4%)	0.0003
1%–49%	22 (10%)	59 (13%)	33 (17%)	44 (10%)	
≥50%	24 (11%)	57 (13%)	24 (12%)	51 (11%)	
Unknown	158 (74%)	293 (66%)	118 (59%)	341 (75%)	
Primary treatment					0.02
Surgery	42 (20%)	127 (29%)	49 (25%)	106 (23%)	
Definitive radiation *	21 (10%)	26 (6%)	11 (6%)	40 (9%)	
Definitive chemoradiation	14 (7%)	34 (8%)	24 (12%)	40 (9%)	
Systemic	59 (28%)	135 (31%)	61 (31%)	110 (24%)	
Symptoms-directed **	68 (32%)	101 (23%)	42 (21%)	134 (30%)	
Unknown	10 (5%)	18 (4%)	13 (7%)	24 (5%)	
Number of investigations per patient ^†^	7.0 ± 2.1	7.3 ± 1.9	6.6 ± 1.6	6.7 ± 1.8	<0.0001
Procedure leading to diagnosis					0.01
Flexible bronchoscopy	55 (26%)	124 (28%)	124 (28%)	134 (30%)	
EBUS/EUS	30 (14%)	84 (19%)	84 (19%)	84 (19%)	
Transthoracic needle biopsy	66 (301%)	97 (22%)	97 (22%)	114 (25%)	
Thoracoscopy	16 (8%)	67 (15%)	67 (15%)	44 (10%)	
Biopsy of a metastasis	27 (13%)	46 (10%)	46 (10%)	55 (12%)	
Thoracentesis	16 (8%)	20 (5%)	20 (5%)	19 (4%)	
Other ^¶^	0	2 (1%)	2 (1%)	2 (0%)	
Unknown	4 (2%)	1 (0%)	1 (0%)	2 (0%)	
Tumor board review	37 (17%)	59 (13%)	30 (15%)	68 (15%)	0.6

^§^ Includes: adenosquamous carcinoma, sarcomatoid carcinoma, carcinoid, large cell carcinoma, neuroendocrine tumor NOS, NSCLC NOS, undifferentiated carcinoma, mixed carcinoma. * Includes stereotactic body radiation therapy (SBRT). ** Includes: palliative radiation, palliative care, no treatment. ^†^ Includes: computed tomography scan (CT-scan), positron emission tomography scan (PET-scan), bone scan, cerebral imaging, abdominal ultrasound, flexible bronchoscopy, EBUS, EUS, transthoracic biopsy, thoracentesis, lymph node biopsy, mediastinoscopy, and biopsy of a metastatic site. ^¶^ Includes: cryoextraction, mediastinoscopy, sputum cytology. Data are expressed as *n* (%) or mean ± SD for dichotomous and continuous variables, respectively, unless otherwise specified. CHUM = Centre hospitalier de l’Université de Montréal; CUSM = Centre universitaire de santé McGill; IUCPQ = Institut universitaire de cardiologie et de pneumologie de Québec; ECOG = Eastern Cooperative Oncology Group; NSCLC = non-small cell lung cancer; SCLC = small cell lung cancer; EGFR = epidermal growth factor receptor; ALK = anaplastic lymphoma kinase; PD-L1 = programmed death-ligand 1; EBUS = endobronchial ultrasound; EUS = endoscopic ultrasound.

**Table 2:** Wait times for investigation and treatment.

Intervals	Time, d
1st abnormal imaging to 1st treatment (*n* = 864)	80 (0–384)
1st appointment with specialist to 1st treatment (*n* = 861)	57 (0–249)
Radiation referral to first radiation treatment (*n* = 188)	29 (0–211)
1st appointment with specialist to surgery (*n* = 304)	76 (6–285)
Diagnosis * to 1st treatment (*n* = 712)	31 (0–185)
Diagnosis to surgery (*n* = 180)	45 (0–185)
Diagnosis to definitive radiation (*n* = 92)	40 (3–168)
Diagnosis to definitive chemoradiation (*n* = 107)	30 (0–118)
Diagnosis to systemic treatment (*n* = 333)	21 (0–138)
Pathology result to biomarkers result (*n* = 378)	14 (0–231)

* Date of diagnosis corresponds to the date the pathology result was reported. Data are expressed as median (range).

**Table 3:** Wait times and overall survival.

Wait Times	*n*	Follow-Up	Unadjusted HR (95% CI)	*p*-Value	Adjusted HR (95% CI) ^†^	*p*-Value
1st abnormal imaging to 1st treatment	776	≤60 days >60 days	0.97 (0.96–0.98) 0.99 (0.99–0.99)	<0.0001 <0.0001	0.98 (0.97–0.99) 1.00 (1.00–1.00)	0.002 0.8
1st appointment with specialist to 1st treatment	767	≤60 days >60 days	0.96 (0.95–0.98) 0.99 (0.99–0.99)	<0.0001 <0.0001	0.98 (0.96–0.99) 1.00 (1.00–1.00)	0.001 0.2
Diagnosis * to 1st treatment	645	≤60 days >60 days	0.92 (0.88–0.95) 0.99 (0.98–0.99)	<0.0001 <0.0001	0.93 (0.89–0.97) 1.00 (1.00–1.00)	0.0002 0.9
Pathology result to biomarkers result	339	≤60 days >60 days	0.95 (0.90–1.01) 1.00 (0.99–1.00)	0.07 0.4	0.95 (0.90–1.01) 1.00 (1.00–1.01)	0.1 0.7

^†^ Adjusted HR for ECOG, histology, and stage as well as primary treatment. * Date of diagnosis corresponds to the date the pathology result was reported. Hazard ratios estimate the change in the risk of death for every added day of waiting time. Because hazard ratios were not proportional over time, values before and after 60 days of follow-up are presented.

**Table 4:** Overall survival model (*n* = 761).

Variables	Univariate Analysis		Multivariate Analysis	
	HR (95% CI)	*p*-Value	HR (95% CI)	*p*-Value
Age	1.01 (1.00–1.02)	0.09	1.01 (1.00–1.03)	0.03
Female sex (vs. male)	0.76 (0.63–0.91)	0.003	0.66 (0.58–0.88)	0.002
Ever-smoker (vs. never)	1.15 (0.77–1.71)	0.5		
ECOG performance status (vs. 0)				
1	2.76 (2.19–3.48)	<0.0001	1.38 (1.07–1.78)	0.01
2	4.30 (3.29–5.63)	<0.0001	1.77 (1.31–2.38)	0.0002
3	8.40 (5.61–12.6)	<0.0001	2.63 (1.63–4.24)	<0.0001
Healthcare network (vs. IUCPQ)				
CHUM	1.00 (0.81–1.25)	1.0		
CUSM	1.04 (0.78–1.37)	0.5		
Alliance Estrie-Montérégie	1.03 (0.78–1.37)	1.0		
Histology and stage (vs. NSCLC stage I)				
NSCLC stage II	2.03 (1.24–3.34)	0.005	2.11 (1.22–3.66)	0.008
NSCLC stage III	5.73 (3.97–8.27)	<0.0001	3.92 (2.40–6.41)	<0.0001
NSCLC stage IV	9.20 (6.50–13.02)	<0.0001	3.61 (1.96–6.65)	<0.0001
LS-SCLC	8.04 (4.89–13.23)	<0.0001	4.44 (2.31–8.54)	<0.0001
ES-SCLC	13.78 (9.32–20.38)	<0.0001	4.46 (2.35–8.47)	<0.0001
Treatment (vs. surgery)				
Definitive radiation *	2.71 (1.81–4.07)	<0.0001	1.67 (1.05–2.66)	0.03
Definitive chemoradiation	4.40 (3.07–6.31)	<0.0001	1.61 (0.99–2.62)	0.06
Palliative systemic treatment	8.49 (6.37–11.31)	<0.0001	2.92 (1.70–4.99)	<0.0001
Wait times **				
1st abnormal imaging to 1st treatment				
≤60 days	0.97 (0.96–0.98)	<0.0001		
>60 days	0.99 (0.99–0.99)	<0.0001		
1st appointment with specialist to 1st treatment				
≤60 days	0.96 (0.95–0.98)	<0.0001		
>60 days	0.99 (0.99–0.99)	<0.0001		
Diagnosis ^†^ to 1st treatment				
≤60 days	0.92 (0.88–0.95)	<0.0001	0.93 (0.89–0.97)	0.0002
>60 days	0.99 (0.98–0.99)	<0.0001		

* Includes SBRT ** Hazard ratios estimate the change in the risk of death for every added day of waiting time. Because hazard ratios were not proportional over time, values before and after 60 days of follow-up are presented. **^†^** Date of diagnosis corresponds to the date the pathology result was reported. ECOG = Eastern Cooperative Oncology Group; IUCPQ = Institut universitaire de cardiologie et de pneumologie de Québec; CHUM = Centre hospitalier de l’Université de Montréal; CUSM = Centre universitaire de santé McGill; NSCLC = non-small cell lung cancer; LS-SCLC = limited-stage small cell lung cancer; ES-SCLC = extensive-stage small cell lung cancer.

## Data Availability

The data presented in this study are available on request from the corresponding author. The data are not publicly available due to confidentiality requirements (personally identifiable information).

## Appendix A

**Table A1:** Wait times for investigation and treatment, NSCLC stages I–II versus III–IV.

Intervals	NSCLC I–II		NSCLC III–IV		*p*-Value
	*n*	Time, d	*n*	Time, d	
1st abnormal imaging to 1st treatment	326	114 (13–384)	413	66 (0–300)	<0.0001
1st appointment with specialist to 1st treatment	319	79 (0–249)	417	50 (0–205)	<0.0001
Radiation referral to first radiation treatment	60	36 (11–145)	107	27 (0–211)	0.5
1st appointment with specialist to surgery	252	77 (6–285)	49	75 (9–205)	0.7
Diagnosis * to 1st treatment	208	48 (0–185)	382	29 (0–147)	<0.0001

* Date of diagnosis corresponds to the date the pathology result was reported. Data are expressed as median (range). NSCLC = non-small cell lung cancer.

**Table A2:** Wait times for investigation and treatment, NSCLC versus SCLC.

Intervals	NSCLC		SCLC		*p*-Value
	*n*	Time, d	*n*	Time, d	
1st abnormal imaging to 1st treatment	739	86 (0–384)	125	34 (2–259)	<0.0001
1st appointment with specialist to 1st treatment	736	62 (0–249)	125	25 (1–191)	<0.0001
Radiation referral to first radiation treatment	167	29 (0–211)	21	35 (14–163)	0.1
Diagnosis * to 1st treatment	590	35 (0–185)	122	13 (0–68)	<0.0001
Diagnosis * to definitive chemoradiation	83	35 (8–118)	24	21 (0–68)	0.0006

* Date of diagnosis corresponds to the date the pathology result was reported. Data are expressed as median (range). NSCLC = non-small cell lung cancer; SCLC = small cell lung cancer.

**Table A3:** Wait times and overall survival, NSCLC stages I–II versus III–IV.

Wait Times	NSCLC I–II			NSCLC III–IV		
	*n*	HR (95% CI)	*p*-Value	*n*	HR (95% CI)	*p*-Value
1st abnormal imaging to 1st treatment	300	1.00 (1.00–1.01)	1.0	368	0.99 (0.99–1.00)	<0.0001
1st appointment with specialist to 1st treatment	292	1.00 (0.99–1.01)	0.9	369	0.99 (0.99–1.00)	<0.0001
Diagnosis * to 1st treatment	194	1.00 (0.99–1.01)	0.6	348	0.99 (0.99–1.00)	0.02

* Date of diagnosis corresponds to the date the pathology result was reported. Hazard ratios estimate the change in the risk of death for every added day of waiting time. NSCLC = non-small cell lung cancer.

**Table A4:** Wait times and overall survival, NSCLC versus SCLC.

Wait Times	NSCLC			SCLC		
	*n*	HR (95% CI)	*p*-Value	*n*	HR (95% CI)	*p*-Value
1st abnormal imaging to 1st treatment	668	0.99 (0.99–0.99)	<0.0001	108	1.00 (0.99–1.00)	0.3
1st appointment with specialist to 1st treatment	661	0.99 (0.99–0.99)	<0.0001	106	1.00 (0.99–1.00)	0.3
Diagnosis * to 1st treatment	542	0.99 (0.99–0.99)	<0.0001	103	0.99 (0.98–1.00)	0.2

* Date of diagnosis corresponds to the date the pathology result was reported. Hazard ratios estimate the change in the risk of death for every added day of waiting time. NSCLC = non-small cell lung cancer; SCLC = small cell lung cancer.
